# Editorial: Development and Application of Novel Genome Engineering Tools in Microbial Biotechnology

**DOI:** 10.3389/fbioe.2020.621851

**Published:** 2020-12-07

**Authors:** Jiazhang Lian, Yi Wang, Yunzi Luo, Chun Li

**Affiliations:** ^1^Key Laboratory of Biomass Chemical Engineering (Education Ministry), College of Chemical and Biological Engineering, Zhejiang University, Hangzhou, China; ^2^Biosystems Engineering Department, Auburn University, Auburn, AL, United States; ^3^Frontier Science Center for Synthetic Biology and Key Laboratory of Systems Bioengineering (Ministry of Education), Collaborative Innovation Center of Chemical Science and Engineering (Tianjin), School of Chemical Engineering and Technology, Tianjin University, Tianjin, China; ^4^Department of Chemical Engineering, Tsinghua University, Beijing, China

**Keywords:** CRISPR-Cas, genome engineering, microbial cell factories, microbial biotechnology, metabolic engineering, synthetic biology

Over the past few years, novel genome engineering tools, especially the CRISPR-Cas system, have emerged and revolutionized our ability to modify microorganisms for both fundamental studies and biotechnological applications. More specifically, recent advances in genome engineering tools have enabled the assembly of multiple and/or large DNA fragments with high efficiency and fidelity, the optimization of biosynthetic pathways in combination and a high throughput manner, and the evolution of microbial cells at an unprecedented speed and scale. Consequently, microorganisms have been extensively engineered to enable us to elucidate microbial cellular machinery and produce renewable fuels and chemicals at high titer, yield, and productivity, or to discover novel natural products as potential drugs. This Research Topic aims to provide an overview of the current status, recent progress, challenges, and future perspectives in the field of microbial genome engineering. It brings together a collection of contributions including original research articles, short communications, reviews, and mini-reviews, from communities involved with genome engineering, metabolic engineering, and microbial biotechnology.

As a revolutionary genome engineering tool, the CRISPR-Cas system has been widely adopted in nearly all kingdoms of life. CRISPR-based multiplex genome engineering tools have been well-established for an increasing list of microorganisms for biotechnological applications. Ding et al. from Beijing University of Chemical Technology provided a comprehensive overview of various CRISPR-Cas tools for microbial genome engineering, including gene activation, gene interference, orthogonal CRISPR systems, and precise single base editing, followed by recent applications in metabolic engineering toward the production of chemicals and natural compounds. Wang K. et al. from Zhejiang University constructed a genetic cassette with triple controls of Cas9 activities at transcriptional, translational, and protein levels, together with the over-expression of the ATP synthase β-subunit AtpD, to achieve efficient genome editing in *Streptomyces*. Zhang J. et al. from Sichuan University developed three efficient CRISPR-FnCas12a systems for multiplex genome editing in several *Streptomyces* strains, among which the CRISPR-FnCas12a1 system was used to efficiently edit the industrial strain *Streptomyces hygroscopicus*, the CRISPR-FnCas12a2 system enabled the deletion of large fragments ranging from 21.4 to 128 kb, and the CRISPR-FnCas12a3 system was employed to successfully recognize a broad spectrum of PAM sequences. Hao et al. from Jiangnan University designed a CRISPR-Cpf1-based toolkit for efficient genome editing in *Bacillus subtili*s, including the flexible deletion of a single gene, multiple genes, a large gene cluster, or gene knock-in. Hu et al. from China Agricultural University report the first application of CRISPR-Cas9 in genome editing of the apicomplexan parasite *Eimeria tenella* for the elucidation of gene functions at the single-gene level as well as for the systematic functional analysis of an entire gene family. Zhou et al. from Shanghai Institutes for Biological Sciences, Chinese Academy of Sciences reported the establishment of a CRISPR-Cas12a-assisted genome editing system in *Amycolatopsis mediterranei*, an industrial producer of rifamycin SV. Kwon et al. from Gyeongsang National University review recent advancements in toolbox development for genome and transcriptome engineering in solventogenic *Clostridium*. Cen et al. from Zhejiang University of Technology reviewed tools in genetic engineering for *Fusarium fujikuroi*, a host for the industrial production of gibberellins. Sun J. et al. from Zhejiang University reported an efficient cytosine base editing system in *Pseudomonas putida*, as well as several other *Pseudomonas* species, without the need for DNA strand breakage and donor DNA templates. Besides genome editing, Zhang K. et al. from Tianjin Institute of Industrial Biotechnology at the Chinese Academy of Sciences established a CRISPR/Cas13d-mediated RNA knockdown platform for *Escherichia coli* and *Corynebacterium glutamicum*.

Of equal importance to the development of genome engineering tools are their applications in the establishment of microbial chassis for the over-production of high value-added compounds, the high level expression of recombinant proteins and enzymes, and the discovery of novel natural products. Zeng et al. from Tianjin University and Liu et al. from Zhejiang University engineered *Saccharomyces cerevisiae* for enhanced production of dihydroartemisinic acid and crocetin, respectively. Xin et al. from Dalian University of Technology employed CRISPR-Cas9 genome engineering tools to delete histidine kinase genes in *Clostridium beijerinckii* for enhanced butanol production. Lo et al. from National Renewable Energy Laboratory utilized the CRISPR-Cas9 system to switch *C. ljungdahlii* from acetogenic to ethanologenic metabolism. Sun Y. et al. from Jiangnan University improved the production of prodigiosin by relieving CpxR temperature-sensitive inhibition in *Serratia marcescens*. Yang et al. from Beijing Institute of Technology reviewed the strategies of optimizing the location and adaptation of pathways on the whole-genome scale for plant natural product biosynthesis in microbial cell factories. In addition to value-added small molecules, Wang Q. et al. from Shanghai Jiaotong University and Zhang T. et al. from Beijing Institute of Technology review the genetic engineering tools developed for the engineering of filamentous fungi in the production of recombinant proteins. Köbbing et al. from RWTH Aachen University systematically characterized the context-dependent effects on synthetic promoters. Lin et al. from Beijing University of Chemical Technology reviewed recent achievements in synthetic biology tools for the cloning of intact natural product biosynthetic gene clusters (BGCs) from complex genome sequences for natural product discovery.

Although the CRISPR-Cas system is now a fashionable technology, other genome engineering tools have applications in microbial biotechnology. Chen et al. from Shenzhen Institute of Advanced Technology in the Chinese Academy of Sciences reviewed recent advances in RNAi-assisted strain engineering in *S. cerevisiae*.

This Research Topic presents a valuable collection of articles focusing on recent developments and applications of novel genome engineering tools for both model (i.e., *E. coli, B. subtilis*, and *S. cerevisiae*) and non-model (i.e., *P. putida, Streptomyces, Clostridium*, and filamentous fungi) microorganisms. As a revolutionary technology, genome engineering and particularly the CRISPR-Cas system, is expected to play increasingly more essential roles in microbial biotechnology as we address the challenges faced by human society.

## Author Contributions

JL drafted the manuscript. YW, YL, and CL read and revised the manuscript. All authors approved the manuscript.

## Conflict of Interest

The authors declare that the research was conducted in the absence of any commercial or financial relationships that could be construed as a potential conflict of interest.

